# Left atrial volume measurement with magnetic resonance imaging: a comparison of biplane, short axis and long axis methods

**DOI:** 10.1186/1532-429X-13-S1-P5

**Published:** 2011-02-02

**Authors:** Kanna Posina, Michael Passick, Nathaniel Reichek, Jie J Cao

**Affiliations:** 1 Saint Francis Hospital, Roslyn, NY, USA

## Introduction

Left atrial (LA) dilation is an important index in cardiovascular disease. The CMR volumetric short axis method is the reference imaging standard but has limited ability to define the mitral valve plane accurately. The biplane method is simpler and frequently employed in clinical settings. We hypothesized that long axis two chamber (2ch) imaging might better define left atrial boundaries.

## Purpose

To compare LA volumes obtained using long axis 2ch stacks to area length biplane and standard short axis methods.

## Methods

Nineteen volunteers (mean age 60.4 ± 14.5) underwent cardiac MRI. SSFP cine imaging with stack of short-axis images across the LA was obtained to determine LA volume using Simpson’s rule. Additionally, a stack of 2ch views along the vertical long-axis plane from the atrial septum to the lateral wall were obtained. Standard 2ch and 4ch cine images were also obtained to determine LA volume by the biplane method. All images were 8 mm with no skip. The biplane method followed the formula: LA Volume= 0.85XA1XA2 /L, where A1 and A2 were areas measured in 2ch and 4ch views, respectively. L was the linear measurement acquired in two different ways, parallel to atrial septum or perpendicular to mitral annulus. Inter and intra-observer variability was determined in 5 randomly selected cases.

## Results

The average LA volume was 69.1±19.6 ml from short axis, 69.1±18.0 ml from 2ch stack, 61.4±21.9 ml from biplane parallel methods, and 67.5±24.1 ml from the biplane method. Bland-Altman analysis showed a mean difference of 0.01±5.5 ml between short axis and 2ch stack volumes. Biplane parallel results differed from short axis and 2ch results by means of 7.7±13.9 ml and 7.6±14.3 ml respectively while biplane perpendicular results differed by 1.6±14.9 ml and 1.6±15.6 ml respectively. Intra-observer variability of 2ch stack and biplane perpendicular methods showed the highest concordance correlation coefficients (CCC) 0.99, followed by the biplane parallel (CCC=0.98) and short axis methods (CCC=0.82). Inter-observer variability showed a similar trend with CCC 0.92-0.95 for 2ch stack, biplane perpendicular, and parallel methods while the short axis had the lowest CCC at 0.78.

## Conclusions

LA volume calculation from long axis 2ch stacks is an excellent alternative to the short axis method with improved reproducibility, likely due to clearer definition of the plane of the mitral annulus. For the biplane method, annular perpendicular length is preferable to septal parallel length.

